# The Therapeutic Effects and Possible Mechanism of Pranoprofen in Mouse Model of Corneal Alkali Burns

**DOI:** 10.1155/2020/7485912

**Published:** 2020-04-06

**Authors:** Minting Chen, Abdirahman Abdinasir Gureeye, Yacouba Cissé, Lang Bai

**Affiliations:** Department of Ophthalmology, Nanfang Hospital, Southern Medical University, Guangzhou, China

## Abstract

**Objective:**

To investigate therapeutic effects and possible mechanism of pranoprofen in a mouse model of corneal alkali burns and provide new evidence for the clinical treatment of corneal alkali burns.

**Methods:**

A unilateral alkali burn was created in the central cornea by placing a piece of 2 mm diameter filter paper soaked in 1N NaOH on the right eye for 30 seconds. After the model was performed, C57BL/6J mice received topical treatment with saline eye drops or pranoprofen eye drops and were, respectively, categorized as saline group and pranoprofen group, whereas the remaining normal mice that were not subjected to alkali burns served as control, each group containing 15 mice (*n* = 45). On the 5th day after model establishment, the corneal fluorescein sodium staining score was evaluated in order to assess corneal epithelial damage. Tissue HE stain was used to observe the pathological changes of corneal tissue in each group. Real-time RT-PCR and western blot were also performed to detect the mRNA and protein expression of NLRP3, IL-1β/*p*17, and matrix metallopeptidase MMP-13.

**Results:**

5 days after burns, microscopic observations of the pranoprofen group showed less corneal opacity and neovascularization development than the saline group. Sodium fluorescein staining showed obvious corneal structure disorders, poor corneal epithelium continuity, and a larger corneal epithelial defect area in the saline group (10.33±+−0.57) as opposed to the pranoprofen group (8.33 ± 0.57） (*p* < 0.05). HE stain results showed the saline group had obvious corneal structure disorder and the corneal epithelial layer was incomplete as opposed to the pranoprofen group. PCR and western blot results suggested that the pranoprofen group expressed less NLRP3, IL-1*β*, and MMP-13 mRNA and protein expression in corneal tissue than the saline group (*p* < 0.05).

**Conclusion:**

Pranoprofen may alleviate inflammatory response by inhibiting the expression levels of NLRP3 and IL-1*β* at the early stage of corneal alkali injury, lowering the expression of MMP-13 and ultimately reducing corneal epithelial damage.

## 1. Introduction

Ocular chemical burns, considered as the main ophthalmic emergencies that require immediate assessment and intensive care, account for approximately 11.5%–22.1% of all ocular traumas [1]. The vast majority of the injuries occur in the workplace as a result of industrial accidents. A minority of injuries occur in the home or secondary to assault. Alkali materials are found more commonly in building materials and cleaning agents and occur more frequently than acid injuries [2]. Alkali agents are lipophilic and therefore penetrate tissues more rapidly than acids [3, 4]. They possess the ability to saponify the fatty acids of cell membranes, penetrate the corneal stroma, and denature the structure of proteins, which results in cell decomposition and necrosis of eye tissues [5]. The damaged tissues then secrete proteolytic enzymes, which lead to severe ocular complications such as perforation, synechia, and disfigurement [6]. Present treatment measures include early irrigation, use of nutrients for cornea and artificial tears, antibiotics, cycloplegic agents, ophthalmic steroid, ascorbic acid, and surgical treatment. In addition to early irrigation in order to block the continuous damage of alkaline substances on the corneal surface, actively reducing inflammation response is one of the important measures to control this condition.

Pranoprofen is a nonsteroidal anti-inflammatory drug (NSAID), widely used in the treatment of inflammation and pain of different origins. However, in ophthalmology department, it is often used for symptomatic treatment of anterior segment's inflammation, such as blepharitis, conjunctivitis, keratitis, and scleritis. Furthermore, nonsteroidal anti-inflammatory drugs (NSAIDs) inhibit cyclooxygenase-1 (COX-1) and COX-2 enzymes, thus blocking arachidonic acid converted to eicosanoids and then reducing the production of prostaglandins [7, 8].

According to various reports, nucleotide-binding oligomerization domain-containing protein (NOD)-like receptor family pyrin domain-containing 3 (NLRP3) inflammasome can induce activation and maturation of caspase-1 precursor and then cleave proinflammatory cytokines IL-1*β* and IL-18 precursors into activated forms, playing an important role in inflammatory response. They have also previously been reported to participate in the occurrence and development of corneal alkali burns, dry eyes, macular degeneration, and other eye diseases [9–11]. Recent studies on whether blocking or inhibiting the activation of the NLRP3 inflammasome through a nonsteroidal anti-inflammatory drug (fenamate) may be essential in memory loss protection in a mouse model of Alzheimer's disease have shown encouraging results [12]. In this study, while exploring pranoprofen therapeutic effects, we found out a new pathway by which it might inhibit alkali burn-induced corneal inflammatory reaction in mice.

## 2. Materials and Methods

### 2.1. Experimental Animals and Grouping

Forty-five healthy female C57BL/6J mice, weighing approximately 20–25 g, were provided by the Experimental Animal Center of Southern Medical University and divided equally among three groups, each group containing 15 mice: mice that were not subjected to alkali burns were classified as the control group, those treated with saline solution were classified as the saline group, and the pranoprofen group designates mice treated with pranoprofen. The experiment was performed on the right eyes, and the left eyes were not treated.

### 2.2. Establishment of a Mouse Model of Corneal Alkali Burns

All experimental procedures were conformed to the ARVO Statement for the “Use of Animals in Ophthalmic and Vision Research” and were approved by the Medical Ethics Committee of Southern Medical University.

Mice were anesthetized by intraperitoneal injection of 1% pentobarbital sodium at a body weight of 5 ∗ 10^−3^ mL/g, and ocular surface anesthesia was performed with 0.5% tetracaine hydrochloride （Santen Pharmaceutical, Japan). The filter paper with a diameter of 2 mm was immersed in a 1 mol/L NaOH solution, and the excess water was absorbed and placed in the center of the right cornea of the mouse for 30 s. After 30 s, the filter paper was removed and eyes were washed with a large amount of physiological saline for 1 min. After the model was established, mice that received saline eye drop treatment, 3 times per day, were classified as the saline group, those who were treated with pranoprofen eye drops (Shandong Haishan Pharmaceutical Co., Ltd.), 3 times per day, were classified as the pranoprofen group, and mice that were not subjected to alkali burns served as the control group.

### 2.3. Sodium Fluorescein Staining and Scoring

Mice were selected from all 3 groups, and sodium fluorescein detection filter paper (Tianjin Jingming New Technology Development Co., Ltd.) was then placed in the conjunctival sac of mice for 3 s. The fluorescein sodium was evenly distributed on the ocular surface through the eyelid closure movement. The corneal staining of the mice was observed under a cobalt blue microscope. According to the four quadrants of the cornea, score 0, no staining; 1 point, punctate staining <30; 2 points, punctate staining >30, but not fusion; 3 points, severe diffuse staining but no plaque staining; and 4 points, large plaque staining [13].

### 2.4. Corneal Tissue HE Stain

3 mice in each group were sacrificed by cervical dislocation, and the eyeballs were immersed in fixative solutions for 24 hours. Later, the eye balls were subjected to tissue dehydration-transparent-dip wax-tissue embedding, tissue sectioning, and then subjected to steps of rehydration-staining-dehydration-sealing, and finally the staining result is observed under a microscope.

### 2.5. Real-Time RT-PCR Detection of Relative Expression of NLRP3, IL-1*β*, and MMP-13 in Corneal Tissue

The mRNA expression of NLRP3, IL-1*β*, and MMP-13 in retrieved corneas was detected by performing real-time RT-PCR on day 5. The corneas were placed in a 1.5 ml centrifuge tube, and total RNA was extracted according to the HiPure Total RNA Plus Mini Kit (Magen). One microgram of total RNA was reverse transcribed using the PrimeScript™ RT reagent kit with gDNA Eraser (Takara Biotechnology Co.). The used primer pair sequences were synthesized by TSINGKE Biological Technology (Beijing, China) and are listed in Table 1. qPCR was performed using SYBR® Premix Ex Taq™ II (Takara Biotechnology Co.) with a QuantStudio5 quantitative PCR instrument (Applied Biosystems). Transcript quantification was performed in triplicate for each sample. Results were reported relative to the control group after normalized by GAPDH using the △△CT method.

### 2.6. Western Blot Detection of NLRP3, IL-1*β*, and MMP-13 Protein Expression in Corneal Tissue

Corneas were washed with cold PBS and cut into pieces, and total protein was extracted according to the Total Protein Extraction Kit (KeyGEN BioTECH, Jiangsu, China). The protein concentration of each group was determined using BCA standards (Leagene Biotechnology, Beijing, China). Proteins (30 mg) extracted from the corneas from each group were subjected to electrophoresis on a 10% or 12% SDS-PAGE and then transferred onto PVDF membranes. The membranes were blocked by incubation with 5% BSA in Tris-buffered saline containing Tween 20 TBST for 1 h at room temperature and incubated overnight at 4 °C with specific rabbit polyclonal antibodies to NLRP3 (1 : 800, Affinity Biosciences), IL-1*β*/*p*17 (1 : 1000Affinity Biosciences), MMP-13 (1 : 500, Cell Signaling Technology), and *β*-actin (1 : 5000, Affinity Biosciences). *β*-Actin was used as internal reference. The membranes were washed three times and incubated for 1 h at room temperature with an HRP-linked goat antirabbit or antimouse secondary antibody (1 : 8000, Affinity Biosciences), followed by visualization of the bands with a chemiluminescence system (Affinity Biosciences). All images were captured and analyzed using a Tanon 5200 Chemiluminescent Imaging System (Tanon Science and Technology Co., Ltd, Shanghai, China), and densitometry analysis of the images was performed using ImageJ software (NIH, USA). The expression level of the above proteins was normalized to those of *β*-actin in the same samples.

### 2.7. Statistical Analysis

Statistical data were analyzed using SPSS 20.0 statistical software and Graphpad Prism 5 software. The relevant indicators were used to represent the mean between groups. The Levene test was used to check and compare the homogeneity of variance. If the homogeneity of variance was satisfying, the comparison between groups was then later analyzed by the *t*-test or one-way analysis of variance. If it is not satisfying, the correction *t*-test or nonparametric test is then used. When *P* < 0.05, the difference is statistically significant.

## 3. Results

### 3.1. Stereoscopic Microscope Observation

**Figure 1:**
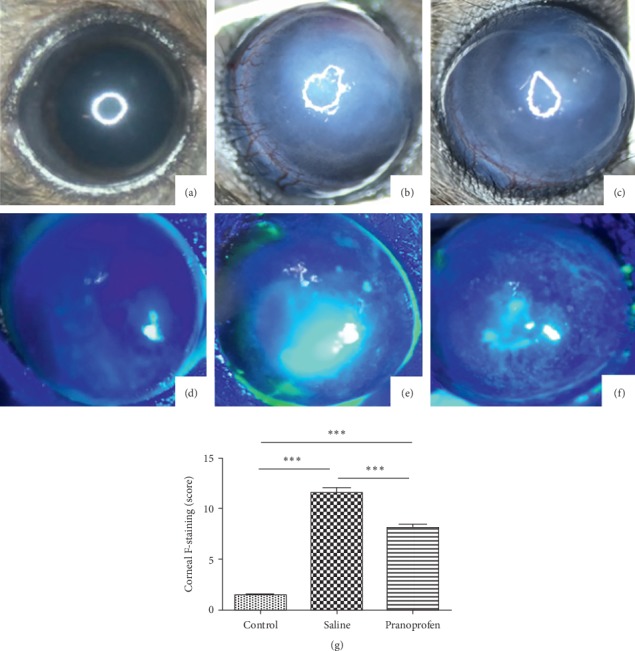
Stereoscopic microscope observation. (upper images) Stereomicroscopic observations on a 5th day in, respectively, control group (a), saline group (b), and pranoprofen group (c). Corneal fluorescein sodium staining and scoring. Staining for corneal fluorescein sodium in the (d) control group, (e) saline group, and (f) pranoprofen group, respectively. (g) Corneal fluorescein staining scores. Data are shown as mean ± SD; ^*∗∗∗*^*P*  <  0.001.

Immediately after the model was performed, corneal opacity was observed in the center of the cornea. As the inflammatory response worsened, the corneal edema aggravated the opacity, the iris texture was unclear, and the neovascularization gradually grew from the limbus to the center. The following pictures are taken five days after burns.  Figure 1(a) shows the stereoscopic observations of the control group, in which the cornea is transparent and the pupil is clearly visible; Figure 1(b) shows the saline group, in which the cornea is obviously edematous, the pupil is invisible, and there is obvious neovascularization; Figure 1(c) shows the pranoprofen group, in which the central cornea is turbid and the irisis still visible.

### 3.2. Corneal Fluorescein Sodium Staining and Scoring

The results of sodium fluorescein staining are shown in Figure 1(d) (control group), Figure 1(e) (saline group), and Figure 1(f) (pranoprofen group) 5 days after burns. No obvious staining was observed in the control group as opposed to the other two groups. Compared to the pranoprofen group, the spots appeared to be more colored, more diffuse, and the plaque-like staining area was also greater in the saline group. Semiquantitative analysis of corneal fluorescein staining scores showed that mean scores of the saline group (11.58 ± 0.49) and the pranoprofen group (8.17 ± 0.34) were significantly higher than the control group (1.50 ± 0.19) （Figure 1(g), ^*∗∗∗*^*P* < 0.001）.

### 3.3. Corneal Tissue HE Stain Results

**Figure 2:**
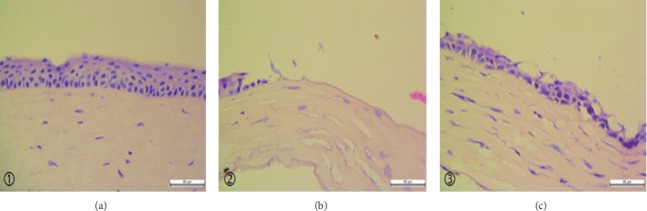
Hematoxylin-eosin staining in cornea. The results of corneal HE staining in the (a) control group, (b) saline group, and (c) pranoprofen group (x400 times, 50 *μ*m).

5 days after burns, the corneas of each group were taken for HE stain (Figure 2). The corneal structure of the control group was clear, and the corneal epithelium was intact. The saline group had obvious corneal structure disorder, and the corneal epithelial layer was missing. In the pranoprofen group, the corneal structure showed more disorders compared to the control group, which was still clearer than the saline group, and the corneal epithelium was also incomplete.

### 3.4. Relative mRNA Expression of Cornea

**Figure 3:**
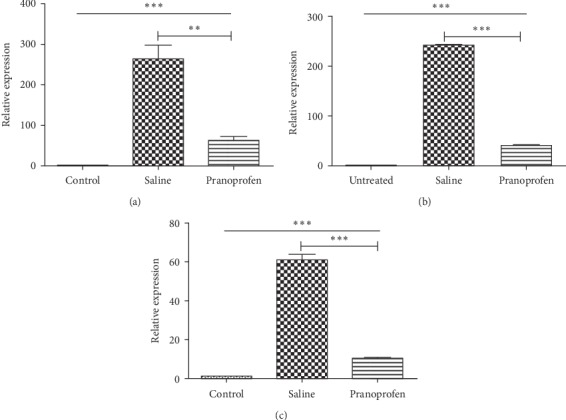
The mRNA expression of NLRP3, IL-1β, and MMP-13 in all groups of cornea. The control group was used as the reference. (a) The mRNA expression of NLRP3 in the pranoprofen group (60.80 ± 10.61) was lower than that of the saline group (264.40 ± 33.45). (b) The mRNA expression of IL-1*β* in the pranoprofen group (40.41 ± 0.50) was lower than that of the saline group (241.30 ± 2.56). (c) The mRNA expression of MMP-13 in the pranoprofen group (10.07 ± 0.22) was lower than that of the saline group (60.81 ± 2.73). Data are shown as mean ± SD; *n* = 6, ^*∗∗*^*P* < 0.01, ^*∗∗∗*^*P* < 0.001.

5 days after burns, compared with the control group, the expression of NLRP3 mRNA in the saline group and the pranoprofen group increased significantly, and the difference was statistically significant (*P* < 0.001) (Figure 3). Compared with the saline group, the expression of NLRP3 mRNA was significantly decreased in the pranoprofen group, and the difference was statistically significant (*P* < 0.01). Compared with the control group, the expression of IL-1*β* mRNA in the saline group and the pranoprofen group increased significantly, and the difference was statistically significant (*P* < 0.001). Compared with the saline group, IL-1*β* mRNA expression was significantly decreased in the pranoprofen group, and the difference was statistically significant (*P* < 0.001). Compared with the control group, the expression of MMP-13 mRNA in the saline group and the pranoprofen group increased significantly, and the difference was statistically significant (*P* < 0.001). Compared with the saline group, MMP-13 mRNA expression was significantly decreased in the pranoprofen group, and the difference was statistically significant (*P* < 0.001).

### 3.5. Relative Protein Expression of Cornea

**Figure 4:**
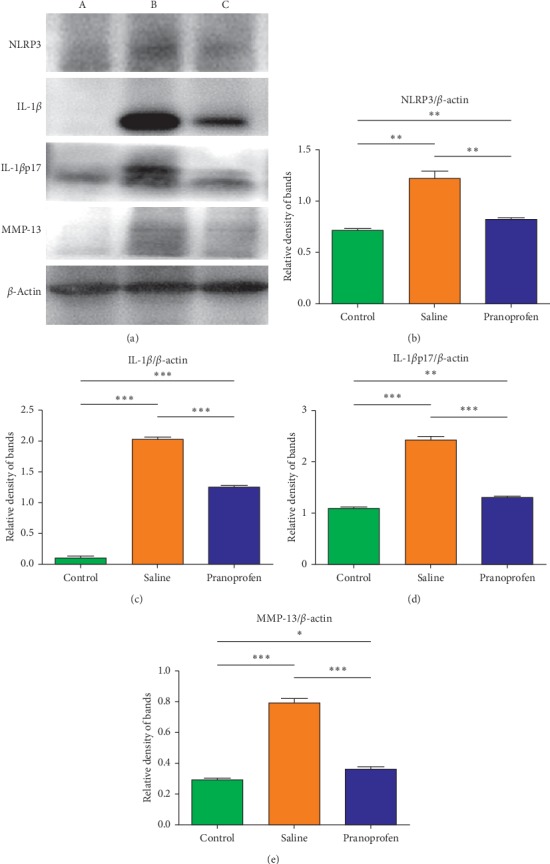
The protein expression of NLRP3, IL-1*β,* IL-1β17p, and MMP-13 in the control group (A), saline group (B), and pranoprofen group (C) by performing western blot. The proteinic expression of (a) NLRP3, (b) IL-1*β*, IL-1*β*17p, and (c) MMP-13 in the control group (0.72 ± 0.024, 0.11 ± 0.002, 1.10 ± 0.028, and 0.30 ± 0.008) appeared to be significantly lower than the other two groups. Additionally, the protein expression of NLRP3, IL-1*β*, IL-1*β*17p, and MMP-13 of the saline group (1.23 ± 0.066, 2.03 ± 0.043, 2.45 ± 0.05, and 0.80 ± 0.030) were higher than those of the pranoprofen group (0.84 ± 0.008, 1.27 ± 0.022, 1.30 ± 0.020, and 0.36 ± 0.017). Data are shown as mean ± SD; *n* = 6, ^*∗*^*P* < 0.05, ^*∗∗*^*P* < 0.01, ^*∗∗∗*^*P* < 0.001.

5 days after burns, the expression levels of NLRP3, IL-1*β*/*p*17, and MMP-13 in the cornea of the saline group and the pranoprofen group were increased compared to the control group. However, the NLRP3, IL-1*β*/*p*17, and MMP-13 proteins in the pranoprofen group were lower than those in the saline group, as shown in Figure 4.

## 4. Discussion

In this experiment, at 5 days after burns, corneal opacity and formation of new blood vessels could be observed under the microscope, corneal epithelial defects can be seen by sodium fluorescein staining, and corneal epithelial defects could be noticed by HE staining; the PCR and western blot detected NLRP3, IL-1*β*, and MMP-13 increased expression. These findings are consistent with reports in the relevant literature. Microscopic observations of the pranoprofen group showed less corneal opacity and neovascularization development than the saline group. Sodium fluorescein staining showed obvious corneal structure disorders, poor corneal epithelium continuity, and a larger corneal epithelial defect area in the saline group, as compared to the pranoprofen group. HE stain results showed that the saline group had obvious corneal structure disorder, and the corneal epithelial layer was incomplete as compared to the pranoprofen group. PCR and western blot results suggested that the pranoprofen group expressed less NLRP3, IL-1*β*, and MMP-13 mRNA and protein expression in corneal tissue than the saline group. The above results suggest that the nonsteroidal anti-inflammatory drug pranoprofen can inhibit the NLRP3 inflammasomes and reduce the release of inflammatory cytokines IL-1*β*, thereby reducing the inflammatory response after corneal alkali burns, and may prevent further damage to the corneal epithelial cells. In addition, pranoprofen can also inhibit the expression of MMP-13, which is closely related to corneal neovascularization after burns.

McCulley divided the clinical course of chemical alkali burns into four phases according to the tissue reaction after acid-alkali burns: immediate phase, acute reparative phase (0–7 days), early reparative phase (7–21 days), and late reparative phase (>3 weeks) [3]. A large number of research studies have proved to be necessary in improving the cure rate of corneal alkali burns to actively prevent the release of inflammatory factors and the infiltration of inflammatory cells in the early stage of burns through the whole course of alkali burns. Effectively preventing the release of proinflammatory cytokines and the infiltration of inflammatory cells at the early stage of alkali burns is the key to improve wound healing in a corneal alkali burn [14, 15]. Inflammasomes are the main multiprotein complex in the cytosol and play a crucial role in innate immunity [16]. Among them, the NLRP3 inflammasome is one of the most widely studied inflammasomes. Its agonists include pathogen-related molecular model PAMPs, such as bacteria, fungi, viruses, and damage-related molecular model DAMPs, such as extracellular ATP, hyaluronic acid, and some environmental stimulants, such as silicon and asbestos. There are still many controversies about the activation pathways of the NLRP3 inflammasome, among which mitochondria release ROS, DNA, cardiolipin, intracytoplasmic potassium ion outflow, and cathepsin release after destabilization of lysosomal membrane are currently the three most recognized pathways [17–19]. The NLRP3 inflammasome can induce caspase-1 precursor maturation and activation, then cleave proinflammatory cytokines IL-1*β* and IL-18 precursors to become activated forms, and secrete them to extracellular participate in inflammatory response [20, 21].

According to various literature reports and combined with previous experimental results, NLRP3 and IL-1*β* expression began to rise within 2 days after alkali burns and peaked or began to decline on day 5. The main differences may be due to the differences of animal model species, duration of burns, ambient temperature, and humidity of selected experimental animals. Therefore, the 5th day was chosen as the termination time in this experiment. Compared with the control group, the real-time RT-PCR results and western blot results in the saline solution group showed that the expression levels of NLRP3 and IL-1*β* were significantly increased, which was consistent with the literature reports. It further indicated that the NLRP3 inflammasome plays an important role in the inflammatory reaction after corneal alkali burns.

For a long time, the main mechanisms of NSAIDs are inhibition of cyclooxygenase-1 (COX-1) and cyclooxygenase-2 (COX-2). By inhibiting COX, arachidonic acid is prevented from converting into eicosanoids, thus reducing the production of proinflammatory prostaglandins [8, 22]. However, with the deepening of research, it has been found that NSAIDs can not only promote inflammation through the COX system but also directly inhibit the production of related inflammatory mediators through non-COX dependence. Michael et al. found that NSAIDs can reduce the production of IL-1*β* by inhibiting the NLRP3 inflammasome in order to alleviate Alzheimer's disease [23]. In addition, there are related reports that NSAIDs not only prevent inflammation by inhibiting the cyclooxygenase pathway but also have anti-inflammatory effects by inhibiting other related molecular targets [24]. Therefore, we believe that the anti-inflammatory mechanism of NSAIDs has molecular targets independent of cyclooxygenase, and the NLRP3 inflammasome is one of the targets of NSAIDs. Despite the fact that NSAIDs may present different structures, they might still possess similar functions. As a member of NSAIDs，whether pranoprofen has the ability of inhibiting NLRP3 inflammasomes has not been reported. Therefore, one of the research objectives of this study is to investigate whether pranoprofen can inhibit inflammation by inhibiting the NLRP3 inflammasome pathway.

Matrix metalloproteinase (MMP), named for its need for Ca2+ and Zn2+ plasma as a cofactor, is mainly responsible for the decomposition of extracellular matrix. Relevant studies suggest that a variety of matrix metalloproteins are involved in the formation of corneal neovascularization, such as MMP-2, MMP-9, and MMP-13. MMP-13 promotes corneal neovascularization by degrading collagen fibers of the extracellular matrix [25–29]. MMP-13 also plays a regulatory and potent role in wound healing, angiogenesis, inflammation, and tumor progression in many normal and pathological processes. Corneal stromal cells are the main cells expressing MMP13 in the corneal stroma after alkali burns. They can directly degrade type I collagen, forming a matrix space and promoting the formation of neovascularizations [29–32]. There is very little literature on the relationship between MMP-13 and corneal inflammation and neovascularization. In this study, we not only observed an upregulation of the expression of MMP-13 postinduction of corneal alkali burns but also found that its expression decreased after pranoprofen application, which may be related to the inflammatory reaction and angiogenesis after corneal alkali burns.

This experiment is only a preliminary exploration of the inhibitory effect of pranoprofen at the early stage of corneal inflammation after alkali burns. There are still many shortcomings in the experiment. Our experiment illustrated the role of the NLRP3 inflammasome in corneal alkali burns. Based on this result, we speculate that effectively inhibiting the expression of the NLRP3 inflammasome in the early stage of alkali corneal burns can inhibit IL-1*β* secretion and reduce the inflammatory response. However, the underlying mechanism about activating and regulating the NLRP3 inflammasome in corneal alkali burns still needs further study. We have speculated from existing reports that pranoprofen has the effect of inhibiting the NLRP3 inflammasome and confirmed this at the molecular level. However, due to only one observation point was selected in our experiment and the lack of observation points in the middle and late stages of burns, the long-term effect of pranaprofen on corneal alkali burns cannot be reflected in this experiment. Additionally, the original purpose of this research was to observe the inhibitory effect of pranaprofen on related inflammatory factors at the early stage of corneal alkali burns, and due to our reduced observation indicators (such as the assessment of corneal neovascularization and function of limbal stem cells), further research is needed in order to fully investigate the inhibition role played by pranoprofen in the inhibition of neovascularization, formation, and some other potential effects after corneal alkali burns.

## 5. Conclusion

In this experiment, pranoprofen application at the early stage of corneal alkali burns led to the reduction of IL-1β production by inhibiting the expression of NLRP3 inflammasomes, which is beneficial in reduction of inflammation response after alkali burns. In addition, pranoprofen can also inhibit the expression of MMP-13, which is closely related to corneal neovascularization after burns. Pranoprofen is a commonly used anti-inflammatory drug in the ophthalmology department. This experiment provides new evidence for its application to corneal alkali burns. However, after corneal alkali injury, there are many factors involved in tissue inflammation and neovascularization, and the mechanism is complex. Therefore, further mechanism research and drug experiments are still needed to explore the disease process and effective treatment in order to attenuate inflammation response, possibly prevent the development of neovascularization, and improve the prognosis of alkali burns.

## Figures and Tables

**Table 1:** Primers for each gene.

Gene name	Sequence of primers (5′–3′)
GAPDH	F: CGTGGAGTCTACTGGTGT
	R: TGTCATATTTCTCGTGGT

NLRP3	F: ATTACCCGCCCGAGAAAGG
	R: TCGCAGCAAAGATCCACACAG

IL-1*β*	F: GAAATGCCACCTTTTGACAGTG
	R: TGGATGCTCTCATCAGGACAG

MMP-13	F: TTCTGGTCTTCTGGCACACG
	R: AGCTCATGGGCAGCAACAAT

## Data Availability

The data used to support the findings of this study are available from the corresponding author upon request.
